# Efficacy of Alveolar Ridge Preservation in Periodontally Compromised Molar Extraction Sites: A Systematic Review and Meta-Analysis

**DOI:** 10.3390/jcm13051198

**Published:** 2024-02-20

**Authors:** Melissa Rachel Fok, George Pelekos, Lijian Jin

**Affiliations:** Division of Periodontology and Implant Dentistry, Faculty of Dentistry, The University of Hong Kong, Hong Kong SAR, China

**Keywords:** periodontitis, periodontally compromised, alveolar ridge preservation, extraction, molar, systematic review

## Abstract

Aim: To investigate the efficacy of alveolar ridge preservation (ARP) in periodontally compromised molar extraction sites. Methods: An electronic search was performed on 10th November 2023 across five databases, seeking randomised/non-randomised controlled trials (RCTs/NCTs) that included a minimum follow-up duration of four months. The RoB2 and Robins-I tools assessed the risk of bias for the included studies. Data on alveolar ridge dimensional and volumetric changes, keratinized mucosal width, and need for additional bone augmentation for implant placement were collected. Subsequently, a meta-analysis was carried out to derive the pooled estimates. Results: Six studies were incorporated in the present review, and a total of 135 molar extraction sockets in 130 subjects were included in the meta-analysis. ARP was undertaken in 68 sites, and 67 sites healed spontaneously. The follow-up time ranged from 4 to 6 months. The meta-analysis of both RCTs and NCTs showed significant differences in mid-buccal ridge width changes at 1 mm level below ridge crest with a mean difference (MD) of 3.80 (95% CI: 1.67–5.94), mid-buccal ridge height changes (MD: 2.18; 95% CI: 1.25–3.12) and volumetric changes (MD: 263.59; 95% CI: 138.44–388.74) in favour of ARP, while the certainty of evidence is graded low to very low. Moreover, ARP appeared to reduce the need for additional sinus and bone augmentation procedures at implant placement with low certainty of evidence. Conclusions: Within the limitations of this study, alveolar ridge preservation in periodontally compromised extraction sites may, to some extent, preserve the ridge vertically and horizontally with reference to spontaneous healing. However, it could not eliminate the need for additional augmentation for implant placement. Further, longitudinal studies with large sample sizes and refined protocols are needed.

## 1. Introduction

Periodontitis is characterized by the destruction of tooth-supporting structures. As periodontally involved teeth progress to a terminal state, extraction becomes inevitable. One of the significant consequences of tooth extraction is the resorption and remodelling of the alveolar ridge, thereby seriously complicating the subsequent placement of dental implants and/or prosthetic rehabilitation [1]. The modelled alveolar ridge may turn atrophic and necessitates complex pre-prosthetic sinus and bone augmentation (BA) procedures, especially at posterior sites that are anatomically delineated by the sinus on the maxillae and the inferior alveolar nerve on the mandible [1,2,3,4,5]. 

In recent years, alveolar ridge preservation (ARP) techniques with various biological materials have been proposed in an attempt to minimise the post-extraction alterations and maintain the ridge dimensions. A recent systematic review and meta-analysis conducted by Couso-Queiruga et al. reported pooled estimated mean for vertical mid-facial, mid-lingual, and horizontal ridge reductions assessed radiographically in molar sites to be 1.46 mm (95% CI: 0.73–2.20), 1.20 mm (95% CI: 0.56–1.83), and 3.61 mm (95% CI: 3.24–3.98), respectively, following spontaneous healing after extraction [6]. The magnitude of post-extraction ridge width reduction was found to be higher in molar than non-molar sites (3.61 vs. 2.54 mm) [6]. Although there is extensive research on the effectiveness of alveolar ridge preservation (ARP) in single-rooted extraction sites in the anterior region with intact sockets, studies focusing on damaged sockets, molar sites, and host-associated factors are very few [7,8,9,10,11,12,13,14,15,16]. The literature reveals considerable variation between individuals in terms of tissue formation and maturation [17]. Multiple factors, such as those related to the patient, procedure, and site, can interact and influence the temporal histogenesis of repair and topographical and dimensional changes following tooth extraction [18,19,20]. Some studies have found that smoking and poorly controlled diabetes can negatively affect the healing process after extraction [21,22,23,24,25,26,27], implying a connection between individual susceptibility and outcomes. However, the specific factors influencing bone physiology and extraction healing remain incompletely understood and are rarely discussed in the literature. The extent of ridge defect as a result of periodontal destruction was listed as a case complexity grade modifier in the most recent 2017 classification for periodontitis, categorising moderate-to-severe ridge defect to more severe form of Stage III/IV periodontitis [28], highlighting the necessity for tailored approaches in managing this particular group of patients. Recently, Atieh and colleagues published a systematic review of ARP in the extraction sockets of periodontally compromised teeth. The study demonstrated short-term beneficial outcomes on alveolar ridge height and bone volume, as well as a decreased necessity for further augmentation procedures [29]. However, the included studies in that systematic review pooled data from all tooth positions and could not find the positive effects of ARP on ridge width changes. Only one of the five included studies by Cha et al. reported results specifically for molar teeth. Notably, the reasons for extraction reported by Cha et al. are not exclusively related to periodontal issues in periodontal patients; they also involve teeth affected by cracks and endodontic failure [3]. Host-related factors were also not clear due to the limited number of included studies. It is noted that ARP studies often excluded smokers and patients with systemic conditions that could potentially compromise healing or bone metabolism. 

Intrinsic anatomical differences between the anterior and molar segments will contribute to different alveolar modelling patterns that call for an independent assessment of the molar extraction sites. Considering the plausibility of the potential effectiveness of ARP on molar sockets affected by periodontal damage and the emerging number of studies in this area, an updated systematic review with meta-analysis is sought. 

## 2. Materials and Methods

### 2.1. Protocol Registration and Reporting Format

This current review adhered to the PRISMA 2020 guidelines. PRIMSA 2020 checklist and PRISMA 2020 abstract checklist are attached in Supplementary Materials. The protocol has been registered in the Open Science Framework (OSF) database hosted by the Center for Open Science. The registered protocol can be publicly accessed at https://osf.ir/mqfhj. Ethics approval was not required for this systematic review.

### 2.2. Objectives

The primary objective of this systematic review was to address the focus question: “What is the efficacy of ARP following the single extraction of a periodontally compromised molar with reference to spontaneous healing?”.

### 2.3. PICOT Question

In order to address the designated focus question, the subsequent study population, intervention, comparison, outcome, and time framework (PICOT) were established and are as follows:

Population (P): adults of ≥18 years, requiring single molar extraction for periodontal reasons.
Intervention (I): alveolar ridge preservation following extraction.
Comparison (C): spontaneous healing following extraction.
Outcome (O): The primary outcomes include changes in alveolar ridge width and height. Secondary outcomes include volumetric change, profilometric change, keratinized mucosal width change, feasibility for implant placement, needs for additional augmentation for implant placement, and patient-reported outcome measures.
Time (T): at least 4-month post-extraction spontaneous healing or following-up of test group intervention.

### 2.4. Information Sources and Search Strategy

To identify ongoing studies with relevant results for inclusion, six databases were systematically searched, i.e., Medline via Ovid, Embase via Ovid, Cochrane Library CENTRAL, Web of Science, The System for Information on Grey Literature in Europe (Opengrey.eu) (access on 10 November 2023), and ClinicalTrials.gov (access on 10 November 2023). The search protocol adhered to the standard procedures outlined by Higgins et al., 2021 [30]. No restrictions pertaining to time or the type of publication and language were imposed during the initial search. On 10 November 2023, the literature search was carried out using a combination of controlled vocabulary and free keywords (Table A1). A manual search of the bibliographies of all included studies and pertinent systematic reviews was conducted to identify additional reports. The comprehensive search strategy for each database can be found in Table A1.

### 2.5. Eligibility Criteria

#### 2.5.1. Inclusion Criteria

This review included human prospective randomised controlled clinical trials (RCTs) and non-RCTs (NCTs), with a minimum of 10 subjects by comparing the ARP method with spontaneous healing, following the extraction of a single molar for terminal periodontitis. The included studies should report on at least one of the following outcomes: changes in alveolar ridge width and height, volumetric change, profilometric change, keratinized mucosal width change, feasibility for implant placement, need for additional sinus procedures or BA for implant placement, and patient-reported outcome measures. No restrictions pertaining to language or publication status was imposed.

#### 2.5.2. Exclusion Criteria

Case reports, abstract only, protocols, in vitro studies, book chapters and proceedings, reviews, animal or cadaver studies, and those without a valid control group were excluded.

### 2.6. Selection of Studies

Using the Covidence platform (Covidence systematic review software, Veritas Health Innovation, Melbourne, Australia, available at www.covidence.org), three previewers (M.F., J.T., and A.K.) independently and in duplicate reviewed the titles, abstracts, and keywords from the search results. Duplicates were eliminated both manually and through the platform’s automated detection and removal feature. The full texts of the remaining articles were then obtained. To identify research articles that fulfilled the eligibility requirements specified in Section 2.5, two reviewers (M.F. and B.C.) independently evaluated the full texts. During the selection process, any disagreements regarding eligibility were resolved via open discussion among the reviewers, with a third reviewer (J.T./B.C.) involved until consensus was achieved. Ineligible articles were excluded, with reasons for exclusion documented. Kappa statistics were used to calculate and report the inter-examiner agreement.

### 2.7. Data Extraction

An electronic data extraction sheet was created using Excel 2019 software. A single reviewer (M.F.) was responsible for the initial data extraction from all the included studies, while a second reviewer (A.K.) verified and checked all the proceedings. Discrepancies in the extracted data were resolved through discussion until a consensus was reached. For each study, the following information was recorded: author, publication year, study design, participant characteristics, age, the number of patients/sites, smoking habits, intervention details, tooth position, socket type, assessment method, post-operative antibiotic use, changes in ridge width and height, volumetric changes, alterations in keratinised mucosal width, necessity for additional sinus procedures or bone augmentation during implant placement, implant success and survival rates, and study follow-up duration.

### 2.8. Quality Assessment and Risk of Bias

Two reviewers (M.F. and J.T.) independently evaluated the methodological quality of the included studies at the study level, with disagreements resolved through discussion. The risk of bias for randomised clinical trials was assessed using the Cochrane risk-of-bias tool for randomised trials (RoB2) [31]. For non-randomised clinical control trials, the ROBINS-I tool [32] was employed.

### 2.9. Statistical Analysis

Meta-analyses for comparable studies that reported the same outcome measures were performed with statistical software package Stata version 16 (StataCorp, College Station, TX, USA). For studies that did not report outcome measurements in mean and standard deviation [33], the estimated mean and standard deviation of the sample were computed according to Luo et al. and Wan et al. [34,35] by extracting the reported sample size, minimum, median, and maximum values. Continuous data, encompassing alterations in ridge vertical and horizontal dimensions, volume, and keratinized mucosal width, were presented as mean differences (MD) with 95% confidence intervals (CIs). Categorical data, including the need for additional sinus procedures and BA during implant placement, were denoted as risk ratios with 95% confidence intervals (CIs). Due to anticipated heterogeneity between studies, the frequentist (classical) random effect approach, specifically the DerSimonian and Laird (DL) method, was employed to combine the results from multiple studies. Owing to the small number of studies incorporated, the fixed effect model (inverse variance (IV) method) was utilized as a sensitivity analysis.

All included studies have the parallel-group design; hence, no statistical adjustments were needed. A formal assessment of publication bias was not conducted since the number of included studies was fewer than 10, resulting in limited power to detect bias in such cases [30]. Each individual site was treated as the statistical unit for analysis purposes. The heterogeneity among studies was evaluated using a Chi-square-based Q-statistic approach and the I-squared metric. An I-squared value exceeding 40% or a *p*-value for the Chi-squared test below 0.10 will be considered as the evidence of significant heterogeneity between studies [36]. Metaregression analysis was performed to investigate study-level factors that drive the measures of effect.

### 2.10. Certainty Assessment

The reliability of the evidence was evaluated following the GRADE criteria, including the risk of bias, inconsistency, imprecision, indirectness, and publication bias [30]. The certainty of evidence for the primary outcomes was assessed and reported with reference to template generated by the GRADEpro software programme (GRADEpro Guideline Development Tool software, McMaster University and Evidence Prime, 2021, available from gradepro.com, access on 17 December 2023).

## 3. Results

### 3.1. Included Studies

**Figure 1:**
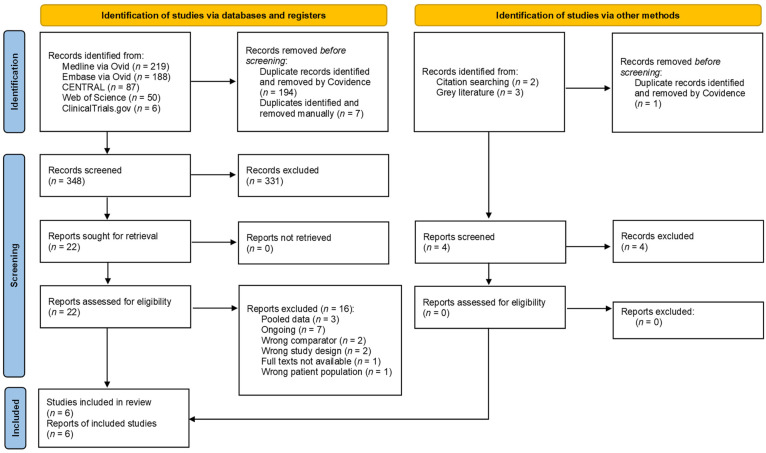
Flowchart illustrating the search strategy and selection process (PRISMA2020).

In total, 555 articles were retrieved from six databases until 10 November 2023. Following the removal of duplicates, 353 articles were screened for their title and abstract, leaving 22 for full-text examination. Sixteen articles were excluded as they failed to meet the eligibility requirements, resulting in the inclusion of six articles for analysis (Figure 1). The reference lists of the included studies were manually searched, but no further articles satisfying the inclusion criteria were identified. During the title and abstract screening process, the Cohen’s Kappa values varied from 0.30 to 0.88 for the three assessors (M.F., J.T., and A.K.), while for the full-text review phase, the value was 0.89 for the two assessors (M.F. and B.C.), demonstrating an excellent agreement.

The study characteristics, participant characteristics, site characteristics, and study intervention are shown in Table 1. Of the 6 included articles, 2 articles [37,38] originated from the same research group and reported different outcomes separately in the 2 articles for the same cohort, resulting in a total number of 5 included studies in this review. Among the 6 included articles, 2 were RCT [2,39] and 4 were non-RCT [33,37,38,40]. 

The studies included were between 2010 and 2023. All studies were conducted in China [33,37,38,39,40] except for one that was conducted in Europe [2]. A total of 130 participants with 135 extraction sites were included, taking note that the 3 articles were under the same clinical trial registration [37,38,40]. All teeth were extracted for advanced periodontal destruction and presented with at least 2 socket walls [2,33,37,38,39,40] with a bone height of at least 3 mm [33,37,38,39,40]. ARP was conducted at 68 sites, while the other 67 sites were allowed to heal naturally after extraction without intervention. Four studies reported healing results at 6 months [2,33,37,40], and one study reported healing results at 4 months [39]. Four studies reported on the need for additional sinus procedures or bone augmentation during implant placement [2,38,39,40]. One study reported implant survival and success up to 36 months [38]. Two studies exclusively studied maxillary molar extraction sites [2,40]. Four studies assessed ridge dimensional changes with CBCT [33,37,39,40], one study additionally assessed ridge height change at the mid-mesial and mid-distal ridge with plain radiograph [33], and one study utilised customised stent with periodontal probe/endodontic file for ridge measurement [2].

### 3.2. Characteristics of Intervention

Prior to ARP, four studies reported performing professional oral hygiene care, with instructions and periodontal treatment to provide an oral environment conducive to wound healing [2,33,37,39]. No information in this regard was available for one study apart from stating the inclusion criteria of patients motivated to have their periodontal disease controlled [40]. Only one included study administered prophylactic antibiotic therapy (Amoxicillin, 1 g, or Erythromycin, 300 mg, if allergic to Penicillin) 1 h before tooth extraction and prescribed 0.2% chlorhexidine rinse for 1 min before the procedure [37]. Local anaesthesia was administrated. Ailing teeth were extracted without trauma and were sectioned with bur when indicated [2,37]. All sockets were debrided. Two studies raised full-thickness flaps, and vertical releasing incisions and performed coronal advancement for primary closure during ARP and reported changes in mid-facial keratinised mucosal width [33,37]. In three studies, full-thickness flaps were not raised, and sites were allowed for secondary intention healing after ARP [2,39,40]. In one study, a full-thickness tunnelling procedure at buccal and lingual ridges to access 2–3 mm of the alveolar bone crest for the application of membrane was performed [40]. Except for one study [39] that filled and overextended autogenous partially demineralised dentin matrix (APDDM) 1–2 mm beyond socket margins and applied a collagen sponge for ARP, the other included studies applied demineralised bovine bone mineral (DBBM) containing products, avoiding the excessive graft outside the confines of the ridge, and overlaid the collagen membrane for ARP. Graft particles were mixed with autogenous blood [33] or saline [37]. One study used a collagen sponge on top of the collagen membrane to seal the site of ARP [40]. All studies prescribed post-operative antibiotics after ARP except for one study where information was unavailable [2]. Non-steroidal anti-inflammatory medications [37,40] and post-operative oral rinsing with 0.12–0.2% chlorhexidine, for 1–2 min, twice a day, for 2–4 weeks were prescribed for the ARP group [33,37,39,40]. Sutures at ARP sites were removed at 2–3 weeks [33,37,39,40]. Prosthesis at the extraction site was prohibited [33,37]. Brushing at the surgical area was avoided for 2 weeks [40]. Weekly recall for the first month and at 3 and 6 months for necessary oral hygiene instructions and periodontal treatment after ARP was documented in one study [37]. 

### 3.3. Clinical Trial Registration

Four studies were registered retrospectively after the initiation of the study [37,38,39,40]. Three of these retrospectively registered studies shared one registration number [37,38,40]. No information on trial registration was available for the two studies [2,33].

### 3.4. Quality Assessment

**Figure 2:**
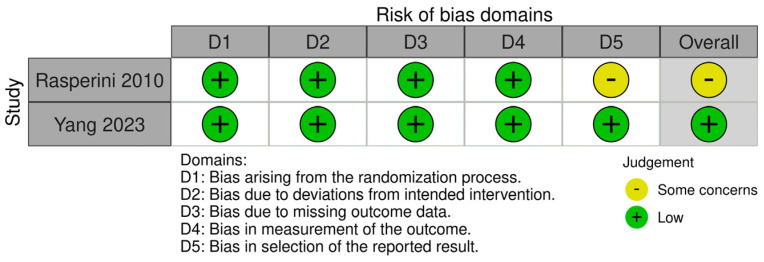
Risk of bias assessment. Quality assessment for RCTs (RoB 2.0).

**Figure 3:**
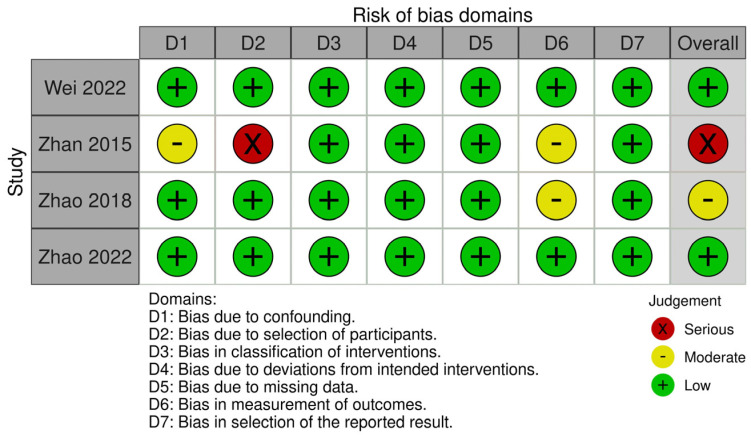
Risk of bias assessment. Quality assessment for non-randomised controlled studies (Robins-I).

Two RCTs were assessed by the RoB2 tool (Figure 2) and presented with low-to-moderate risk. All RCTs reported on the methods of randomisation and adequate allocation concealment. One study only reported one of the listed outcomes at one of the two measured time points (6-month post-healing) and hence was subjected to reporting bias. Four non-randomised controlled studies were assessed with the ROBINS-I tool and were at low-to-severe risk of bias (Figure 3). One study had a serious risk of bias due to the confounding selection of participants according to the treatment needs of and planning for the patient [33]. Two NCT studies had moderate concerns arising from the bias in the measurement of outcomes [33,41].

Three included studies reported on the sample size calculation [37,39,40]. 

### 3.5. Horizontal Dimensional Changes

Ridge width changes were measured at mid-socket, mesial-quarter, and distal-quarter of socket at 1, 3, and 5 mm level below the ridge crest in two studies [37,39], at 1, 4, and 7 mm level below the ridge crest in one study [33], and only at 1 mm level below the ridge crest in one study [40]. Measurements at 3 and 4 mm levels and 5 and 7 mm levels were pooled for meta-analysis. 

**Figure 4:**
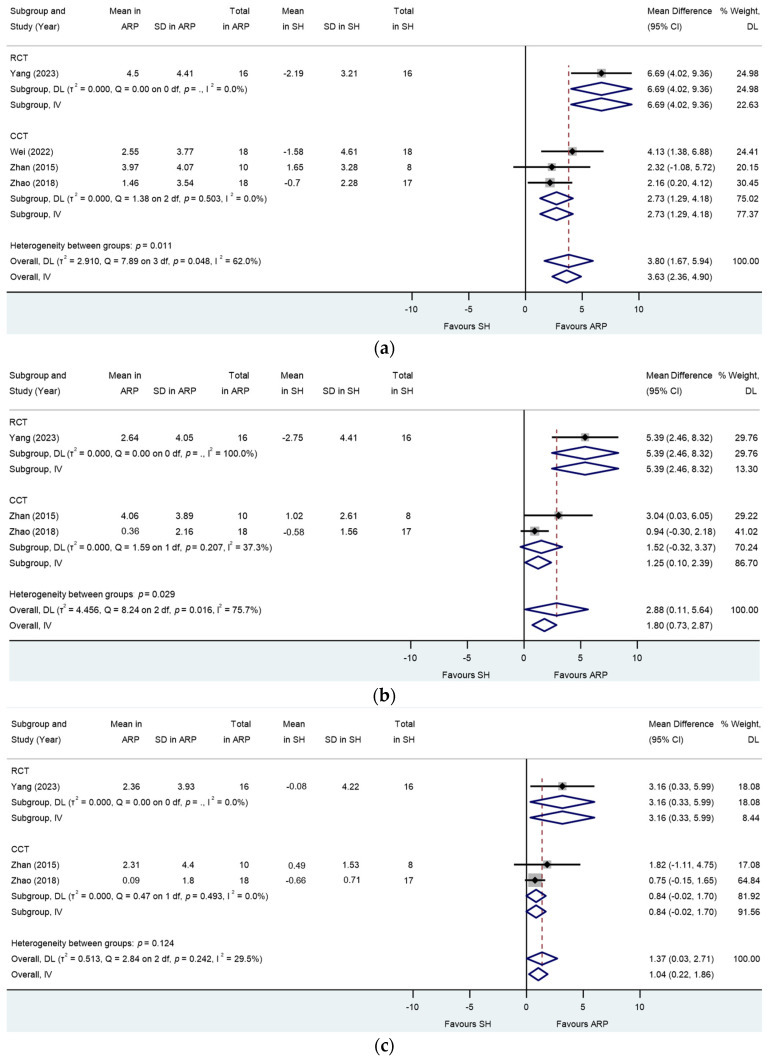
Alveolar ridge preservation versus spontaneous healing. Subgroup analyses (RCTs and CCTs): changes in the width of the alveolar ridge at (**a**) mid-ridge 1 mm level below the ridge crest, (**b**) mid-ridge 3–4 mm level below the ridge crest, and (**c**) mesial-quarter ridge at 3–4 mm level below the ridge crest. *p* = .: *p* value not computed for subgroup.

**Figure A1:**
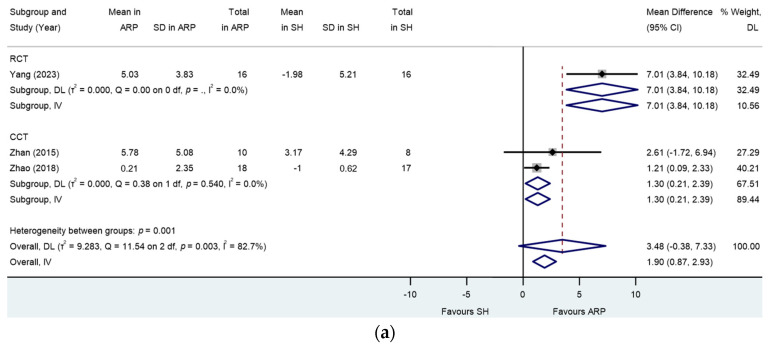

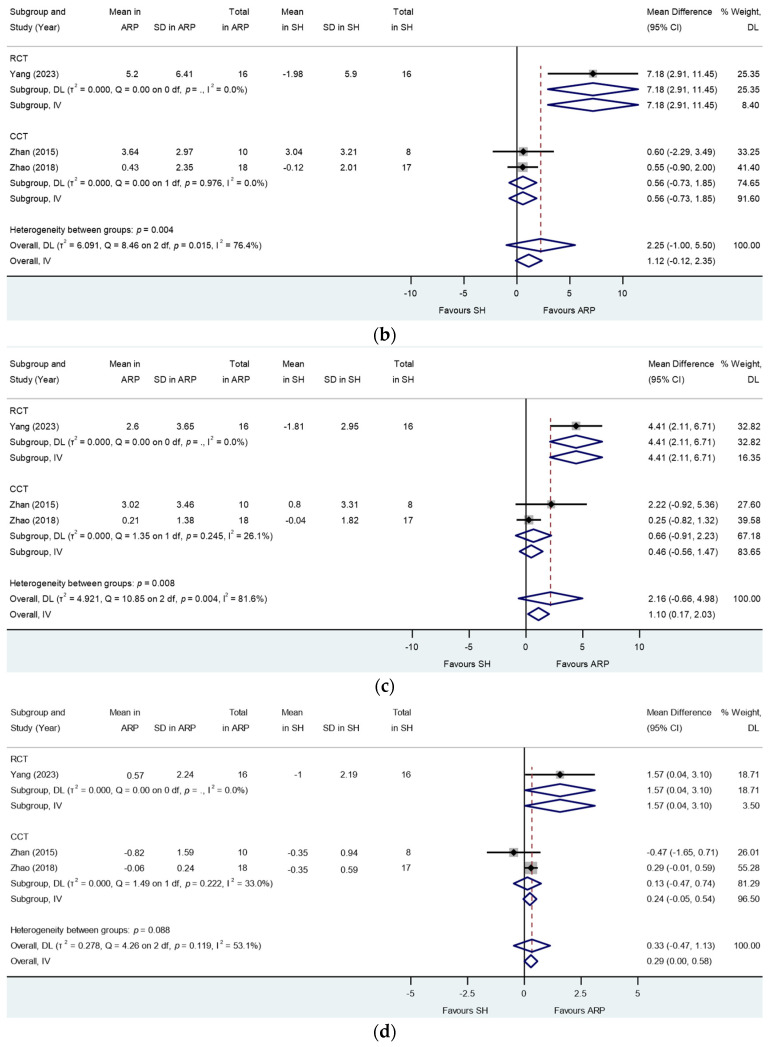

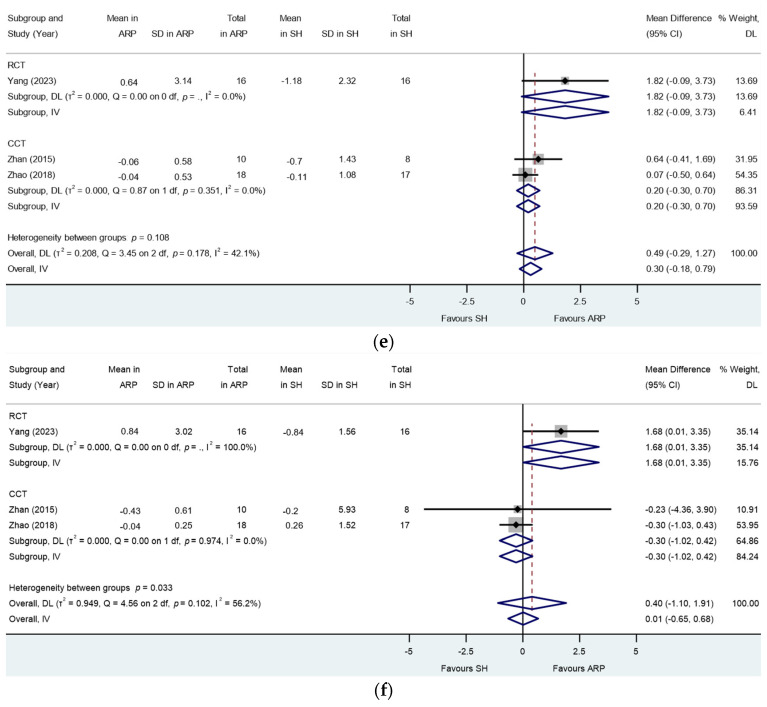
Alveolar ridge preservation versus spontaneous healing. Subgroup analyses (RCTs and CCTs): changes in the width of the alveolar ridge at (**a**) mesial-quarter ridge at 1 mm level below the ridge crest, (**b**) distal-quarter ridge at 1 mm level below the ridge crest, (**c**) distal-quarter ridge at 3–4 mm level below the ridge crest, (**d**) mesial-quarter ridge at 5–7 mm level below the ridge crest, (**e**) mid-ridge at 5–7 mm level below the ridge crest, and (**f**) distal-quarter ridge at 5–7 mm level below the ridge crest. *p* = .: *p* value not computed for subgroup.

When performing meta-analyses in separate and combined RCTs [39] and NCTs [33,37,40], statistically significant and less ridge width reduction was detected at mid-ridge at 1 mm level (MD: 3.80; 95% CI: 1.67 to 5.94; *p* > 0.001; Figure 4a) and 3–4 mm level (MD: 2.88; 95% CI: −0.11 to 5.64; *p* = 0.001; Figure 4b) and mesial-quarter-ridge at 3–4 mm level (MD: 1.37; 95% CI: 0.03 to 2.71; *p* = 0.046; Figure 4c) in the ARP group compared to the SH group. Heterogeneity was detected for analysis at mid-ridge at 1 mm and 3–4 mm levels (I^2^ < 40.00%, *p* > 0.10; Figure 4a,b). No heterogeneity was detected for analysis at mesial-quarter-ridge at 3 mm level (I^2^ < 29.50%, *p* = 0.24; Figure 4c). No statistically significant reduction in ridge width was detected at 5–7 mm level for all locations and the rest of the measured locations (Figure A1).

Meta-regression showed no association of study type (RCT/NCT) with response for analysis at mid-ridge 1 mm level (*p* = 0.71) and 3–4 mm level (*p* = 0.30) and mesial-quarter-ridge at 1 mm level (*p* = 0.37).

### 3.6. Vertical Dimensional Changes

Ridge height changes were measured at nine sites around the extraction site (mid-buccal, mid-oral, mesial-buccal, mesial-oral, disto-buccal, disto-oral, mesial-central, mid-central, and distal-central) in two studies [37,39], excluding mid-central socket measurement at eight sites in one study [33], at mid-buccal, mid-oral, and mid-central sockets in one study [40], and at mid-buccal and mid-central in one study [2]. 

**Figure 5:**
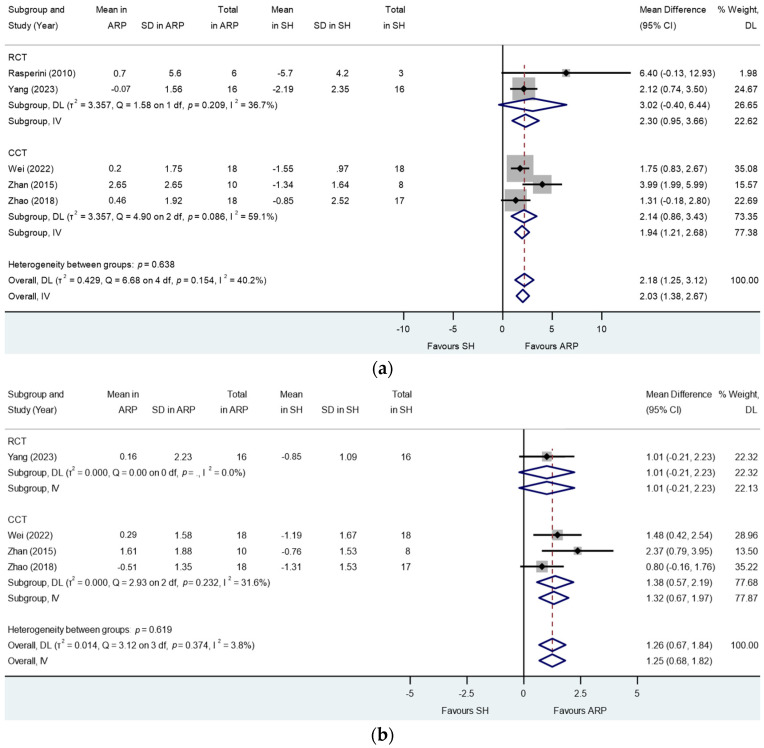
Alveolar ridge preservation versus spontaneous healing. Subgroup analyses (RCTs and CCTs): changes in the alveolar ridge height at (**a**) mid-buccal and (**b**) mid-oral sockets. *p* = .: *p* value not computed for subgroup.

The meta-analysis of RCTs showed a trend towards lesser reduction in mid-buccal ridge height in the ARP group versus the control group while not reaching statistical significance in the random effect model (MD: 3.02; 95% CI: −0.40 to 6.44; *p* = 0.08; Figure 5a). Sensitivity analysis in the fixed effect model showed statistical significance (MD: 2.30; 95% CI: 0.95 to 3.66, 0.34; *p* = 0.001). Heterogeneities were not detected (I^2^ = 36.70%, *p* = 0.21). When combining RCTs and NCTs, the meta-analysis showed statistically significant and lesser reduction in mid-buccal ridge height in the ARP group versus that of the SH group (MD: 2.18; 95% CI: 1.25 to 3.12; *p* < 0.001; Figure 5a). Marginal heterogeneity was detected (I^2^ = 40.20%, *p* = 0.15; Figure 5a). Meta-regression showed no association of study type (RCT/NCT) with response (*p* = 0.80). 

When combining RCTs and NCTs, the meta-analysis showed statistically significant and lesser reduction in mid-oral ridge height in the ARP group versus that of the SH group (MD: 1.26; 95% CI: 0.67 to 1.84; *p* < 0.001; Figure 5b). No heterogeneity was detected (I^2^ = 3.80%, *p* = 0.37). Meta-regression showed no association of study type (RCT/NCT) with response (*p* = 0.71).

**Figure A2:**
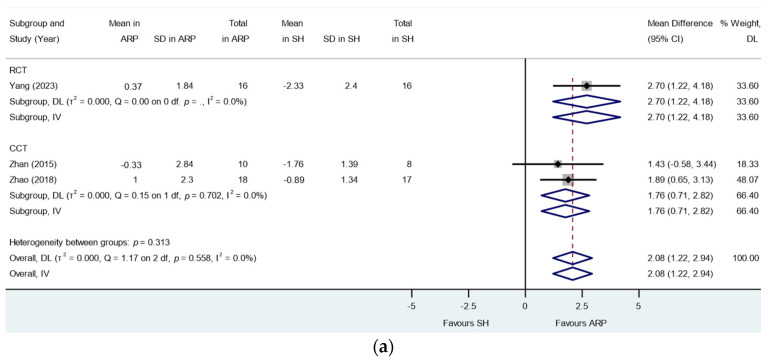

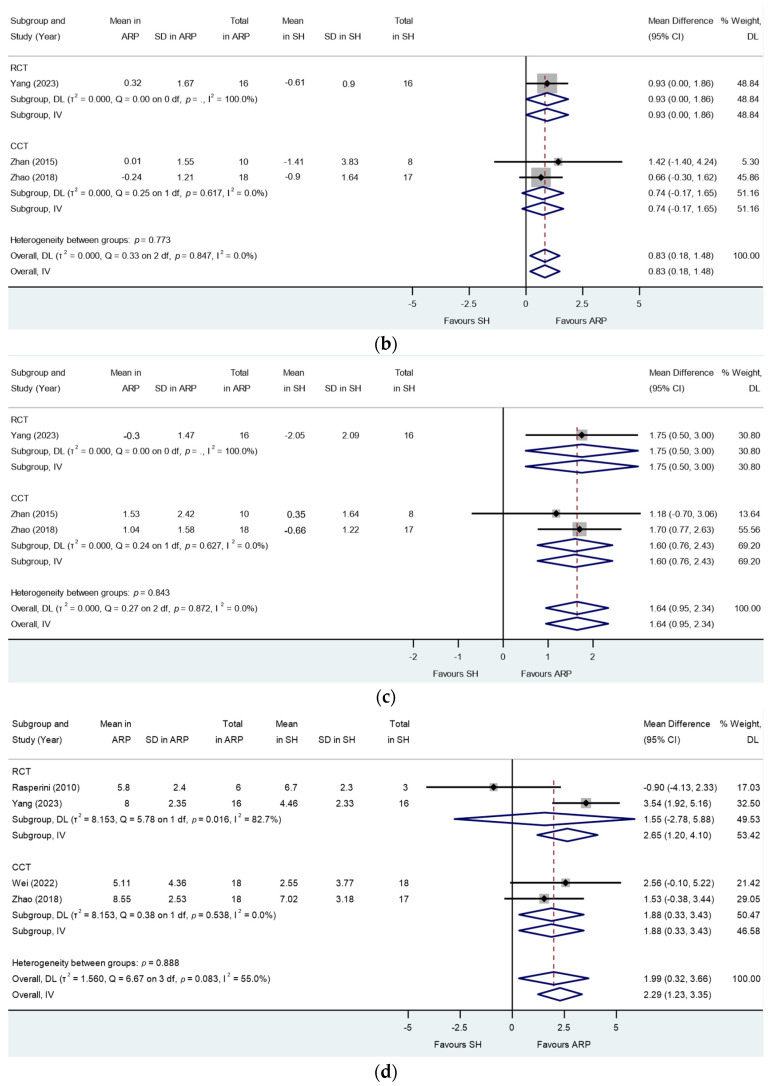
Alveolar ridge preservation versus spontaneous healing. Subgroup analyses (RCTs and CCTs): changes in the alveolar ridge height at (**a**) mesial-buccal, (**b**) mesial-oral, (**c**) disto-buccal, and (**d**) mid-central sockets. *p* = .: *p* value not computed for subgroup.

Similarly, the meta-analysis showed statistical significant and lesser reductions in mesial-buccal (MD: 2.08; 95% CI: 1.22 to 2.94; *p* < 0.001; Figure A2a), mesial-lingual (MD: 0.83; 95% CI: 0.18 to 1.48; *p* = 0.01; Figure A2b), disto-buccal (MD: 1.64; 95% CI: 0.95 to 2.34; *p* < 0.001; Figure A2c), and mid-central (MD: 1.99; 95% CI: −0.32 to 3.66; *p* < 0.001; Figure A2d) ridge heights in the ARP group compared to those of the SH group. No heterogeneity was detected in the above analyses (I^2^ < 40%, *p* > 0.10). 

**Figure A3:**
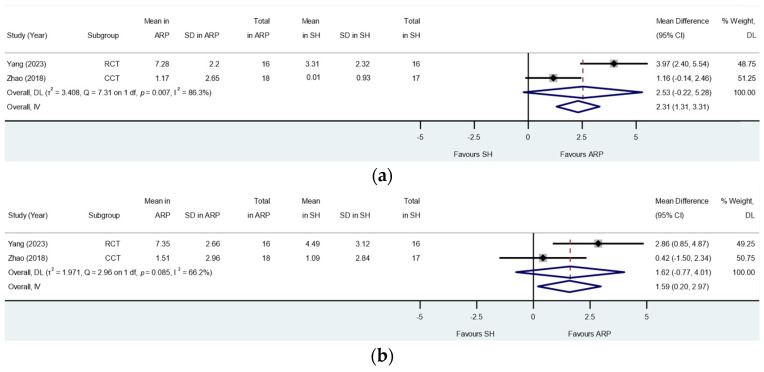

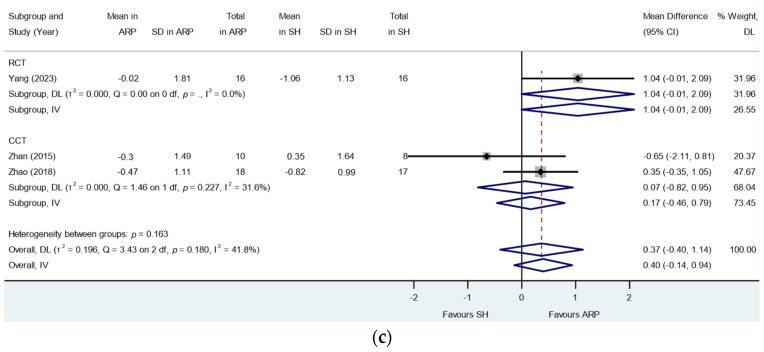
Alveolar ridge preservation versus spontaneous healing. Subgroup analyses (RCTs and CCTs): changes in the alveolar ridge height at (**a**) mesial-central, (**b**) distal-central, and (**c**) distal-oral sockets. *p* = .: *p* value not computed for subgroup.

The meta-analysis showed a trend towards less ridge height reduction with no statistically significant differences in distal-lingual (MD: 0.37; 95% CI: −0.40 to 1.14; *p* = 0.15; Figure A3c), mesial-central (MD: 2.53; 95% CI: −0.22 to 5.28; *p* = 0.07; Figure A3a), and disto-central (MD: 1.62; 95% CI: −0.77 to 4.01; *p* = 0.18; Figure A3b) ridge heights in the ARP group versus those of the SH group without statistical significance. No heterogeneity was detected in the above analyses (I^2^ < 40%, *p* > 0.10). 

### 3.7. Volumetric Changes

**Figure 6:**
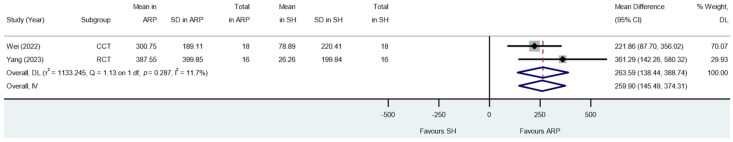
Alveolar ridge preservation versus spontaneous healing. Subgroup analyses (RCTs and NCTs): alveolar ridge volumetric change.

Two studies (one RCT and one NCT) documented alterations in alveolar ridge volume [39,40]. The meta-analysis revealed a statistically significant advantage in the ARP group compared to the control group (MD: 263.59; 95% CI: 138.44 to 388.74; *p* < 0.001; Figure 6). No heterogeneity was detected (I^2^ = 11%, *p* = 0.29).

### 3.8. Keratinized Mucosal Width

**Figure 7:**
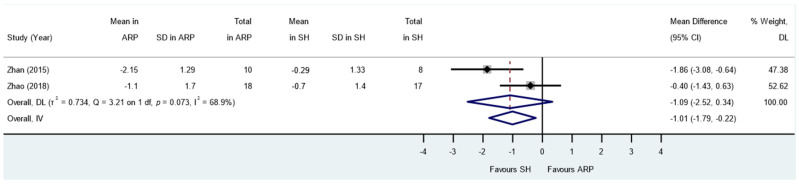
Alveolar ridge preservation versus spontaneous healing. Subgroup analyses (RCTs and CCTs): change in keratinised mucosal width.

Two NCTs reported keratinised mucosal width changes [33,37]. Full-thickness flap elevation with vertical releasing incisions and flaps advancement coronally for primary closure was performed for ARP after graft and membrane insertion. The meta-analysis showed a trend towards more loss of keratinised mucosal width in the ARP group versus the control group while not reaching statistical significance in the random effect model (MD: −1.09; 95% CI: −2.52 to 0.34; *p* = 0.13; Figure 7). Heterogeneity was detected (I^2^ = 68.9%, *p* = 0.07). 

### 3.9. Needs for Additional Augmentation for Implant Placement

**Figure 8:**
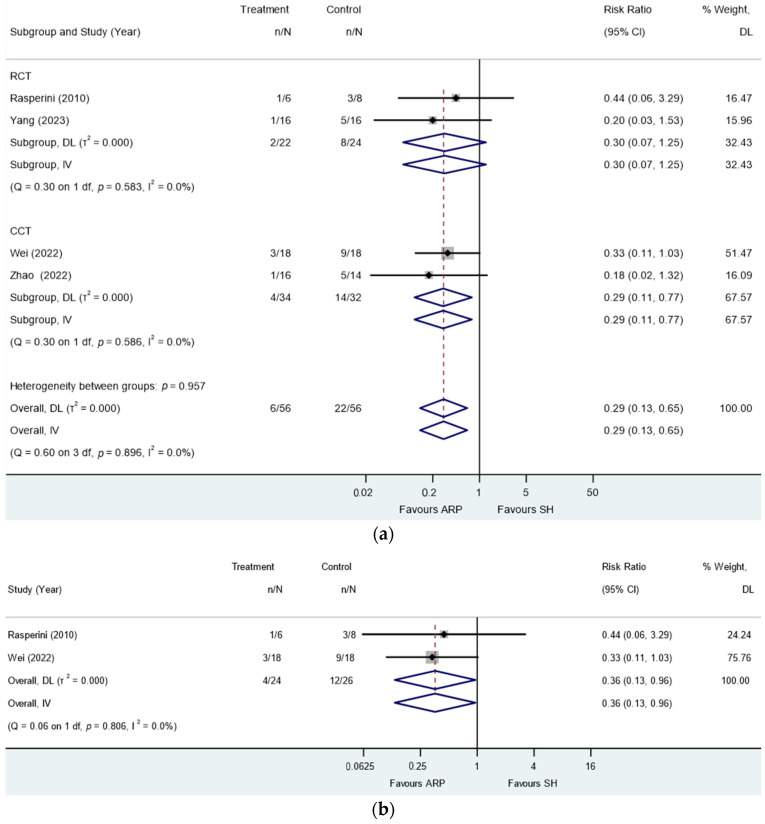
Alveolar ridge preservation versus spontaneous healing. Subgroup analyses (RCTs and CCTs): need for additional sinus procedure or augmentation at the time of implant placement: (**a**) maxillary and mandibular molars and (**b**) maxillary molars only.

Four studies [2,38,39,40] reported the need for additional sinus procedures and BA for implant placement after healing. A total of 112 sites in this review received implants. Among those sites that received implants, 33 and 36 maxillary molar sites received AHP and SH, while 23 and 20 mandibular molar sites received AHP and SH. Overall, 34 out of the 56 SH sites did not require additional sinus procedures or augmentation for implant placement. The meta-analysis revealed that employing ARP considerably decreased the necessity for additional augmentation during implant placement in comparison to the SH group (RR: 0.29; 95% CI: 0.13 to 0.65; *p* = 0.003; Figure 8a). The analysis did not identify any presence of heterogeneity (I^2^ = 0.00%, *p* = 0.90). Meta-regression showed no association of study type (RCT/NCT) with response (*p* = 0.96).

Two of these studies exclusively studied maxillary molar extraction sites [2,40]. In the meta-analysis of the two studies, it was demonstrated that the application of ARP significantly diminished the requirement for additional augmentation during implant insertion in comparison to the SH group (RR: 0.36; 95% CI: 0.13 to 0.96; *p* = 0.04; Figure 8b). The analysis did not identify any presence of heterogeneity (I^2^ = 0.00%, *p* = 0.81).

### 3.10. Implant-Related Outcomes

Only one follow-up study [38] of its parent study, which was also included in this present review [37], reported on the long-term survival and success rates of implants placed in ARP and SH sites. The success criteria set out by Karousis et al., 2004, were followed [42]. The total implant survival rate was 100% in both groups; the success rate was 81.2% in the test group and 78% in the control group at three-year follow-up. No statistically significant differences in marginal bone level changes, modified plaque index, probing depth, and modified sulcus bleeding index were noted in both groups. 

## 4. Discussion

Overall, two RCTs and four NCTs have been included in this systematic review, following a strict selection criterion to minimise heterogeneity and potential bias. The studies were limited to those that only dealt with molar teeth extracted due to terminal periodontitis while excluding other damaged sockets resulting from teeth extracted for other reasons. In a systematic evaluation of the evidence, we compared ARP and SH after the extraction of periodontally compromised teeth, focusing on outcomes such as alterations in alveolar ridge width and height, ridge height modifications, volumetric shifts, changes in mid-facial keratinized mucosal width, the requirement for supplementary sinus procedures or augmentation during implant placement, and implant-related outcomes. Our meta-analysis results have shown that AHP has significantly favourable effects, with low to very low certainty of evidence on (i) preserving alveolar ridge width of damaged extraction sites at the mid-ridge 1 mm and 3–4 mm levels below the ridge crest and mesial-quarter ridge at 3–4 mm level below ridge crest; (ii) preserving alveolar ridge height at mid-buccal, mid-oral, and mesial-buccal sockets; (iii) preserving volume; and (iv) reducing the needs for additional sinus procedure and BA for implant placement.

Over the past decade, there has been a vast collection of systematic reviews on the increasingly hot topic of ARP [7,8,11,29,43], including studies utilising different techniques and grafting materials for APR. The most recent Cochrane systematic review in 2021 reported very-low-certainty evidence on the reduction in the loss of ridge width (MD: −1.35 mm, 95% CI: −2.00 to −0.70) from a meta-analysis of six studies favouring xenograft. However, they could not detect significant differences in the need for additional augmentation or implant failure rate [8]. The majority of the included studies in the Cochrane review excluded molar sites, and even for those studies that included molar sites, the results were either pooled with other tooth types [44,45,46,47] or pooled with teeth without periodontal involvement [3,47,48]. Notably, there exists evidence that the healing profile of sockets from the extraction of periodontally diseased teeth varies, in which osseous regeneration was slower [49], and indeed, the advanced radiographic bone loss of ≥75% significantly prolonged the time of socket cortification [50]. It is known that the periodontally compromised sockets present a reduced number of socket walls and are topographically limited by fewer tissue resources from residual bone walls. Hence, there is less regenerative potential. Given speculation of such difference, a subsequent systematic review specifically studied ARP in the extraction sockets of periodontally compromised teeth [29]. However, three [51,52,53] out of the five included studies are on non-molar teeth, and the remaining two do not have separate analyses for molar teeth [3,54]. Atieh et al. reported very low to low certainty of evidence that ARP may have short-term positive effects on ridge height (MD: −0.95; 95% CI: −1.43 to −0.47; *p* < 0.0001) and volume (MD: −38.70; 96% CI: −52.17 to −25.24; *p* < 0.0001), and reduced need for additional augmentation procedure, which are in general agreement with the results of our present study. The outcome of using ARP in periodontally compromised sites reported in this review is more favourable than that of Atieh et al., 2022. Meta-analysis in our review is also available for up to nine topographical regions of the extraction site where we have identified the main benefit of the procedure at the mid-ridge and mesial-quarter of the ridge. The alveolar ridge at the molar region is anatomically broader as compared to its anterior counterparts. Hence, the relevance of ARP in the posterior molar area has been questioned, especially when an extracted tooth presents minimal clinical attachment loss, an intact alveolus, and good residual basal bone height. The addition of bone substitute materials in the extraction site does not enhance new bone formation [55] and results in lower bone formation than ungrafted sites [56,57], despite being able to contribute to preserving the ridge profile. The justification for ARP with the intention to reduce subsequent surgical complexity in implant rehabilitation appears to be more vital for periodontally damaged maxillary posterior sites and those with limited basal bone height [3]. However, there is a lack of prospective data on whether consecutive molar extractions will lead to aggravated bone atrophy and may benefit more from ARP. If the residual bone volume is judged to be sufficient for early or late implant placement with minor GBR [58] or osteotome sinus floor elevation, or on the contrary, the site is compromised beyond repair, or if ARP complicates prosthetically driven implant treatment planning, there is little reason for the APR procedures. Indeed, a previous ARP study in the anterior region showed similar clinical, aesthetic, and patient-reported outcome measures for early implant placement with or without ARP [59], pointing to the negligible clinical added value and cost–benefit of ARP if early implant placement is feasible. Although no similar studies have been conducted for the posterior region to the best of our knowledge, comparable conclusions might potentially be drawn.

ARP appears to be a relatively safe procedure provided that the infectious source is thoroughly removed. However, failures were reported. Kim et al. in a retrospective study including 297 cases of APR at compromised extraction sockets reported a 97.3% safety rate [60], in which 8 patients developed inflammatory symptoms and 2 patients eventually required biomaterials removal. All their patients were administered antibiotics up to 1 week following ARP. No graft failure was reported from the studies in this review. This may be attributed to the rigorous periodontal disease control regimen provided before and throughout interventions. 

Only two ARP treatment modalities were detected in the present review, while a recent systematic review identified up to nine treatment modalities, including the use of cortico-cancellous porcine bone particles, allograft particles, autologous blood-derived products, and cell therapy for ARP [8]. Further studies on other techniques and methods for ARP in the posterior region would be needed. Interestingly, a recent study [39] contrasted other DBBM ARP studies and instead investigated the use of APDDM processed from the extracted autogenous teeth/third molar for ARP. Only dentine matrix particles of 425 to 1200 μm that met the application-specific standards were used [61]. The graft material was overfilled, and the site allowed for a secondary intention of healing, utilising a collagen sponge as a socket seal. This study resulted in the best ARP outcomes in terms of ridge width and height changes assessed by CBCT. However, it is unclear if the overextended graft particles were well integrated with the coronal portion of the socket at the time of implant placement or otherwise presented fibrous adhesions at the cervical part of previously preserved sockets and merely manifested as a radiopaque mass in the CBCT. The efficacy of APDDM as a bone substitute material for lateral alveolar ridge augmentation and alveolar ridge preservation has been previously demonstrated in both animal and human studies with minimal complications [62,63,64,65,66]. The dentine graft undergoes gradual replacement resorption and supports osseointegration, with a percentage area of vital bone exceeding the area of APDDM at 3.5 months of healing in humans [67]. Following the careful preparation and removal of unwanted tissues, the ankylosis of APDDM to recipient bone can be induced with substitution by vital bone, and APDDM presents an attractive autogenous bone substitute for ARP [67]. However, a substantial chairside processing time is needed for preparation, and inadvertently retained enamel remnants are present. 

The existing evidence implies that performing primary intention wound closure/flap advancement for ARP may not be essential. However, the sockets must be sealed for the perceived benefits of ARP [14]. Various socket sealing strategies for ARP to protect the inserted bone substitute, including flap advancement, the use of collagen membrane and collagen sponge/matrix, and connective tissue graft or free gingival graft, have demonstrated similar outcomes [14]. The results of the present review are consistent with their findings. However, the present review indicates that flap advancement tends to result in a reduction in mid-facial keratinised mucosal width. Balancing the need to maintain procedural effectiveness, streamline the surgical process, maximise keratinised tissue, and minimise patient discomfort after surgery, an open healing with barrier procedure for ARP may be preferred. 

The limitations of this study pertain to the heterogeneity arising from the inclusion of NCTs apart from RCTs, as one of the included studies used a different approach [2] to assess hard tissue changes and different surgical techniques and biomaterials in ARP. Furthermore, data from maxillary and mandibular molar sites were pooled together for analysis in three studies, and the sample size in the studies was small, thereby limiting the ability to perform subgroup analysis by tooth location. Future ARP studies could greatly benefit from the standardization and reporting of various key parameters, such as the type of tooth used, pre/post-operative patient/professional hygiene regimen, flap elevation techniques/healing approach, biomaterials selection, antibiotics prescription, wound care, and follow-up. Further investigations should seek to report patient-reported outcomes, the duration of surgeries, and the cost-effectiveness of the overall ARP and implant procedures, as these important outcomes were not reported in the studies included in the present review. 

## 5. Conclusions

Within the limitations of this study, alveolar ridge preservation in periodontally compromised extraction sites could, to some extent, reduce the vertical and horizontal ridge dimensional alteration with reference to spontaneous healing. However, it may not eliminate the need for additional augmentation during implant placement. Further, longitudinal studies with large sample sizes and refined clinical protocols are highly warranted. 

## Figures and Tables

**Table 1:** Study, subject characteristics, and interventions of the 6 reviewed articles.

		Rasperini et al., 2010 [2]	Yang et al., 2023 [39]	Zhan et al., 2015 [33]	Zhao et al., 2018 [37]	Zhao et al., 2022 [38]	Wei et al., 2022 [40]
**Study Design**		RCT, parallel group	RCT, parallel group	NCT, parallel group	NCT, parallel group	NCT, parallel group	NCT, parallel group
**Region and Setting**		Europe, 2 centres	China, hospital	Beijing, China, hospital	Beijing, China, hospital	Beijing, China, hospital	Beijing, China, hospital
**Number assessed (participants/sites)**		14/14	32/32	16/18	32/35	26/30	36/36
	ARP	6	16	10	18	16	18
	SH	8	16	8	17	14	18
**Ages (years)**	Mean (SD)	54	ARP: 48.56 (13.46)	NR	ARP: 50.40 (8.90)	ARP: 49.90 (7.50)	ARP: 50.5 (8.90)
			SH: 58.94 (16.09)		SH: 49.70 (7.00)	SH: 51.70 (7.10)	SH: 49.80 (9.40)
	Range	NR	ARP: 21–69	NR	ARP: 34–65	NR	ARP: 26–61
			SH: 22–79		SH: 34–59	NR	SH: 30–63
**Smoking patterns, Smoker, *n* (%)**		Non-smoker	Non-smoker, ≤10 cigarettes/day	Non-smoker	Non-smoker, ≤10 cigarettes/day	Non-smoker, ≤10 cigarettes/day	Non-smoker, ≤10 cigarettes/day
	ARP	0	NR	0	0	0	NR
	SH	0	NR	0	0	0	NR
**Intervention**							
	ARP	DBBM with 10% collagen ^a^ and collagen membrane ^b^, and suture with secondary intention healing	Autogenous partially demineralised dentin matrix (APDDM) and collagen sponge ^c^, and suture with secondary intention healing	DBBM ^d^ and collagen membrane ^b^, full thickness flap, one vertical releasing incision for coronal advancement, and primary intention healing	DBBM ^d^ and collagen membrane ^b^, full thickness flap, two vertical incisions for coronal advancement, and primary intention healing	Same as Zhao 2018	DBBM ^d^, collagen membrane ^b^, collagen sponge ^c^, full thickness tunnelling, and secondary intention healing
	SH	Suture only	No sutures	Suture only	Suture only	Same as Zhao 2018	Suture only
**Surgical technique**		Microinvasive extraction, root separation with burs, flapless, and secondary intention healing	Minimal traumatic extraction, flapless, and secondary intention healing	Microinvasive extraction with full-thickness flap and primary intention healing	Atraumatic extraction, root separation with diamond fissure burs if required, full thickness flap, and primary intention healing	Same as Zhao 2018	Atraumatic extraction, full thickness tunnelling, and secondary intention healing
**Teeth**		First or second maxillary molar	Upper and lower molars	Upper and lower molars	Upper and lower molars	Same as Zhao 2018	Upper molars
**Type of socket**		Four-wall and buccal wall damaged sockets	One-wall, two-wall, three-wall, four-wall sockets, defect height > 50% of root length, and at least two walls with bone height of ≥ 3 mm	Two-wall, three-wall, and at least two walls with bone height of ≥ 3 mm	One-wall, two-wall, three-wall, four-wall sockets, and at least two walls with bone height of ≥ 3 mm	Same as Zhao 2018	At least two walls with bone height of ≥ 3 mm
**Method of assessment**		Customised stent with periodontal probe/endodontic file measurement	CBCT	CBCT, parallel periapical radiograph	CBCT	same as Zhao 2018	CBCT
**Post-operative antibiotics**		NR	Cefuroxime 250 mg and Metronidazole 500 mg, tds, 3 days	Amoxicillin 500 mg, tds, 7 days	Unknown antibiotics, tds, 7 days	Same as Zhao 2018	Amoxicillin 500 mg, tds, 7 days/Clarithromycin 250 mg, bd, 7 days
**Changes in ridge width at mid-socket (mm)**							
**ARP**	HW1	NR	4.50 (4.41)	4.80 (−3.50, 9.10)	1.46 (3.54)	NR	2.55 (3.77)
	HW3	NR	2.64 (4.05)	NR	0.36 (2.16)	NR	NR
	HW4	NR	NR	3.50 (−1.30, 11.00)	NR	NR	NR
	HW5	NR	0.64 (3.14)	NR	−0.04 (0.53)	NR	NR
	HW7	NR	NR	−0.10 (−0.90, 0.90)	NR	NR	NR
**SH**	HW1	NR	−2.19 (3.21)	1.45 (−2.80, 6.60)	−0.70 (2.28)	NR	−1.58 (4.61)
	HW3	NR	−2.75 (4.41)	NR	−0.58 (1.56)	NR	NR
	HW4	NR	NR	0.15 (−1.70, 5.80)	NR	NR	NR
	HW5	NR	−1.18 (2.32)	NR	−0.11 (1.08)	NR	NR
	HW7	NR	NR	−0.15 (−3.40, 0.70)	NR	NR	NR
**Changes in ridge width at the mesial-quarter of the socket (mm)**							
**ARP**	HW1	NR	5.03 (3.83)	5.80 (−2.10 13.60)	0.21 (2.35)	NR	NR
	HW3	NR	2.36 (3.93)	NR	0.09 (1.80)	NR	NR
	HW4	NR	NR	0.40 (−1.80, 11.80)	NR	NR	NR
	HW5	NR	0.57 (2.24)	NR	−0.06 (0.24)	NR	NR
	HW7	NR	NR	−0.65 (−3.50, 1.40)	NR	NR	NR
**SH**	HW1	NR	−1.98 (5.21)	2.35 (−2.00, 10.30)	−1.00 (0.62)	NR	NR
	HW3	NR	−0.80 (4.22)	NR	−0.66 (0.71)	NR	NR
	HW4	NR	NR	−0.20 (−0.90, 3.50)	NR	NR	NR
	HW5	NR	−1.00 (2.19)	NR	−0.35 (0.59)	NR	NR
	HW7	NR	NR	−0.10 (−2.00, 0.70)	NR	NR	NR
**Changes in ridge width at the distal-quarter of the socket (mm)**							
**ARP**	HW1	NR	5.2 (6.41)	3.4 (−0.60, 8.60)	0.43 (2.35)	NR	NR
	HW3	NR	2.6 (3.65)	NR	0.21 (1.38)	NR	NR
	HW4	NR	NR	2.35 (−1.40, 9.30)	NR	NR	NR
	HW5	NR	0.84 (3.02)	NR	−0.04 (0.25)	NR	NR
	HW7	NR	NR	−0.35 (−1.50, 0.40)	NR	NR	NR
**SH**	HW1	NR	−1.98 (5.90)	2.90 (−1.40, 7.80)	−0.12 (2.01)	NR	NR
	HW3	NR	−1.81 (2.95)	NR	−0.04 (1.82)	NR	NR
	HW4	NR	NR	−0.10 (−2.80, 6.70)	NR	NR	NR
	HW5	NR	−0.84 (1.56)	NR	0.26 (1.52)	NR	NR
	HW7	NR	NR	0.05 (−9.00, 8.00)	NR	NR	NR
**Changes in ridge height (mm)**							
**ARP**	Mid-buccal	0.7 (5.6)	−0.07 (1.56)	2.90 (−1.80, 6.40)	0.46 (1.92)	NR	0.20 (1.75)
	Mid-oral	NR	0.16 (2.23)	1.55 (−1.20, 4.60)	−0.51 (1.35)	NR	0.29 (1.58)
	Mesial-buccal	NR	0.37 (1.84)	−0.35 (−4.70, 4.10)	1.00 (2.30)	NR	NR
	Mesial-oral	NR	0.32 (1.67)	−0.05 (−2.30, 2.50)	−0.24 (1.21)	NR	NR
	Distal-buccal	NR	−0.30 (1.47)	1.45 (−2.10, 5.40)	1.04 (1.58)	NR	NR
	Distal-oral	NR	−0.02 (1.81)	−0.15 (−2.80, 1.80)	−0.47 (1.11)	NR	NR
	Mesial-central	NR	7.28 (2.20)	0.04 (−1.55, 2.64)	1.17 (2.65)	NR	NR
	Mid-central	5.8 (2.4)	8.00 (2.35)	NR	8.55 (2.53)	NR	5.11 (4.36)
	Distal-central	NR	7.35 (2.66)	2.26 (−0.66, 6.94)	1.51 (2.96)	NR	NR
**SH**	Mid-buccal	−5.7 (4.2)	−2.19 (2.35)	−1.00 (−4.10, 0.60)	−0.85 (2.52)	NR	−1.55 (0.97)
	Mid-oral	NR	−0.85 (1.09)	−0.30 (−3.50, 0.90)	−1.31 (1.53)	NR	−1.19 (1.67)
	Mesial-buccal	NR	−2.33 (2.40)	−1.55 (−4.00, 0.00)	−0.89 (1.34)	NR	NR
	Mesial-oral	NR	−0.61 (0.90)	−1.25 (−7.10, 3.90)	−0.90 (1.64)	NR	NR
	Distal-buccal	NR	−2.05 (2.09)	−1.45 (−13.80, 0.30)	−0.66 (1.22)	NR	NR
	Distal-oral	NR	−1.06 (1.13)	−0.15 (−1.40, 3.30)	−0.82 (0.99)	NR	NR
	Mesial-central	NR	3.31 (2.32)	−0.09 (−1.55, 2.64)	0.01 (0.93)	NR	NR
	Mid-central	6.7 (2.3)	4.46 (2.33)	NR	7.02 (3.18)	NR	2.55 (3.77)
	Distal-central	NR	4.49 (3.12)	−0.18 (−5.70, 3.17)	1.09 (2.84)	NR	NR
**Volumetric changes (mm^3^)**							
	ARP	NR	387.55 (399.85)	NR	NR	NR	300.75 (189.11)
	SH	NR	26.26 (199.84)	NR	NR	NR	74.89 (220.41)
**Mid-facial keratinised mucosal width**							
	ARP	NR	NR	−2.15 (1.29)	−1.1 (1.7)	NR	NR
	SH	NR	NR	−0.29 (1.33)	−0.7 (1.4)	NR	NR
**Needs for additional sinus procedure or augmentation at the time of implant placement (event/total)**							
	ARP	1/6	1/16	NR	NR	1/16	3/18
	SH	3/8	5/16	NR	NR	5/14	9/18
**Implant success (%) (36 months)**							
	ARP	NA	NR	NR	NR	100	NR
	SH	NA	NR	NR	NR	100	NR
**Implant survival (%) (36 months)**							
	ARP	NA	NR	NR	NR	78.60%	NR
	SH	NA	NR	NR	NR	81.20%	NR
**Follow-up period (months)**		6	4	6	6	36	6

Abbreviations: ARP, alveolar ridge preservation; SH, spontaneous healing; CBCT, cone beam computed tomography; NA, non-applicable; NR, not reported; RCT, randomised controlled trial; NCT: non-randomised controlled trial; HW1, 3, 5, 7, changes in horizontal ridge width at 1-, 3-, 5-, 7 mm below the ridge crest. ^a^ Bio-Oss Collagen, Geistlich Pharma AB, Wolhusen, Switzerland. ^b^ Bio-Gide Geistlich Pharma AB, Wolhusen, Switzerland. ^c^ Wuxi BIOT Biological Engineering Co., Ltd., Jiangsu, China. ^d^ Bio-Oss, Geistlich Pharma AB, Wolhusen, Switzerland.

## Data Availability

No new data were created for this study.

## Supplementary Materials

The following supporting information can be downloaded at: https://www.mdpi.com/article/10.3390/jcm13051198/s1.

## Appendix A

**Table A1:** Study, patient characteristics, and interventions of the reviewed studies.

MEDLINE via Ovid (1946 to 10 November 2023, *n* = 219)
#1 ((alveol* adj4 ridge preserv*) or (ridge adj4 preserv*) or (socket adj4 preserv*) or (ridge adj4 augment*)).mp. #2 (molar* or posterior*).mp. #3 ((periodont* adj disease*) or periodont* or (periodont* adj compromised) or (damage* adj4 socket) or dehiscence).mp. #4 #1 AND #2 AND #3
Embase via Ovid (1947 to 10 November 2023, *n* = 188)
#1 ((alveol* adj4 ridge preserv*) or (ridge adj4 preserv*) or (socket adj4 preserv*) or (ridge adj4 augment*)).mp. #2 (molar* or posterior*).mp. #3 ((periodont* adj disease*) or periodont* or (periodont* adj compromised) or (damage* adj4 socket) or dehiscence).mp. #4 #1 AND #2 AND #3
The Cochrane Library CENTRAL (until 10 November 2023, *n* = 87)
#1 (alveol* NEAR/4 ridge preserv*) OR (ridge NEAR/4 preserv*) OR (socket NEAR/4 preserv*) OR (ridge NEAR/4 augment*) #2 molar* or posterior* #3 (periodont* NEAR/4 disease*) or periodont* or (periodont* NEAR/4 compromised) or (damage* NEAR/4 socket) or dehiscence #4 #1 AND #2 AND #3 in Trials
Web of Science (1956 to 10 November 2023, *n* = 50)
#1 TS = ((alveol* NEAR/4 ridge preserv*) OR (ridge NEAR/4 preserv*) OR (socket NEAR/4 preserv*) OR (ridge NEAR/4 augment*)) #2 TS = (molar* or posterior*) #3 TS = (periodont* NEAR/4 disease*) or periodont* or (periodont* NEAR/4 compromised) or (damage* NEAR/4 socket) or dehiscence #4 #1 AND #2 AND #3
Clinicaltrials.gov (until 10 November 2023, *n* = 6)
Intervention/treatment: Alveolar ridge preservation Filter: Adult, Older adult Study type: interventional Study Results: With results
Opengrey (until 10 November 2023, *n* = 3)
((alveol* ridge preserv* OR ridge preserv* OR socket preserv* OR ridge augment*)) AND (molar OR posterior) AND ((periodont* disease) OR (periodont*) OR (periodont* compromised) OR (damage* socket) OR dehiscence)

**Table A2:** Certainty assessment.

Certainty Assessment							№ of Site		Relative Effect	Absolute Effect		Absolute Difference	Certainty	Importance
№ of Studies	Study Design	Risk of Bias	Inconsistency	Indirectness	Imprecision	Other Considerations	ARP	SH	Relative (95% CI)	With ARP	Without ARP			
			Changes in mid-ridge width 1 mm below the ridge crest (mm)											
1	Randomised controlled trial	Not serious	Not serious	Not serious	Concerns due to the small sample number	Publication bias is strongly suspected; plausible residual confounding may reduce the demonstrated effect	16	16	MD 6.69 mm higher (4.02 mm to 9.36 mm higher)	4.5 mm higher	2.19 mm lower	Not estimable	⨁⨁◯◯ Low ^a,d^	High
			Changes in the height of the alveolar ridge at mid-buccal (mm)											
2	Randomised controlled trial	Moderate	Not serious	Not serious	Concerns due to the small sample number	Publication bias is strongly suspected; all plausible residual confounding may reduce the demonstrated effect	22	24	MD 3.02 mm higher (0.40 mm lower to 6.44 mm higher)	0.14 mm higher	2.88 mm lower	Not estimable	⨁◯◯◯ Very Low ^b,d^	High
			Changes in alveolar ridge volume (mm^3^)											
2	Randomised controlled trial and non-randomised controlled trial	Not serious	Not serious	Not serious	Concerns due to the small sample number	Publication bias suspected; all plausible residual confounding may reduce the demonstrated effect	34	34	MD 263.59 mm^3^ higher (138.44 mm^3^ to 388.74 mm^3^ higher)	341.60 mm higher	78.01 mm higher	Not estimable	⨁⨁◯◯ Low ^a,d^	Low
			Needs for additional sinus procedure or augmentation at the time of implant placement (randomised controlled trial)											
2	Randomised controlled trial	Not serious	Not serious	Not serious	Concerns due to the small sample number	Publication bias is strongly suspected; all plausible residual confounding would suggest spurious effect, while no effect was observed	22	24	RR: 0.30 (0.07–1.25)	9.10% 91 in 1000	31.34% 313 in 1000	Risk difference −0.24 (95% CI: −0.46, −0.02) 240 fewer per 1000 patients (95% CI: 20 fewer to 460 fewer). Based on data from 44 patients in 2 studies	⨁⨁◯◯ Low ^a,d^	High
			Needs for additional sinus procedure or augmentation at the time of implant placement (non-randomised controlled trials)											
2	Non-randomised controlled trial	Not serious	Not serious	Not serious	Concerns due to the small sample number	Publication bias is strongly suspected	34	32	RR: 0.29 (0.11–0.77)	11.76% 118 in 1000	40.57% 406 in 1000	Risk difference −0.31 (95% CI: −0.51, −0.11) 310 fewer per 1000 patients (95% CI: 110 fewer to 510 fewer). Based on data from 66 patients in 2 studies	⨁⨁◯◯ Low ^a,d^	High
			Needs for additional sinus procedure or augmentation at the time of implant placement (RCTs and NCTs)											
4	RCTs and NCTs	Not serious	Not serious	Not serious	Concerns due to the small sample number	Publication bias is strongly suspected	56	56	RR: 0.29 (0.13–0.65)	10.71% 107 in 1000	36.74% 367 in 1000	Risk difference −0.28 (95% CI: −0.43, −0.13) 280 fewer per 1000 patients (95% CI: 130 fewer to 430 fewer). Based on data from 112 patients in 4 studies	⨁⨁◯◯ Low ^a,d^	High
			Mid-facial keratinised mucosal width (mm)											
2	Non-randomised controlled trial	Serious	Heterogeneity detected	Not serious	Concerns due to the small sample number	Publication bias is strongly suspected	28	31	1.09 mm lower (from 2.52 mm lower to 0.34 mm higher)	1.48 lower	0.39 lower	Not estimable	⨁◯◯◯ Very Low ^c,e^	High

CI: confidence interval; RR: risk ratio. GRADE Working Group grades of evidence. ⨁ and ◯: They indicate certainty of evidence. The more symbols ⨁, the higher the certainty. High certainty: we are very confident that the true effect lies close to that of the estimate of the effect. Moderate certainty: we are moderately confident in the effect estimate—the true effect is likely to be close to the estimate of the effect, but there is a possibility that it is substantially different. Low certainty: our confidence in the effect estimate is limited—the true effect may be substantially different from the estimate of the effect. Very low certainty: we have very little confidence in the effect estimate—the true effect is likely to be substantially different from the estimate of the effect. ^a^ Downgraded one level: due to suspected risk of publication bias. ^b^ Downgraded two levels: due to the risk of publication bias and one RCT presented moderate concerns arising from bias due to selection of reported results. ^c^ Downgraded three levels: serious risk of bias due to the selection of participants and moderate concern arising from bias due to confounding in one study. Two NCT studies have moderate concerns arising from bias in the measurement of outcomes. ^d^ Downgraded one level: due to imprecision as there are a limited number of studies, and the studies have a small sample number of participants. ^e^ Downgraded one level due to inconsistency: substantial heterogeneity was detected; *p* = 0.073; I^2^ = 68.9%.
